# 1-Methyl-2-({[(2-methyl­phen­yl)meth­yl]disulfan­yl}meth­yl)benzene

**DOI:** 10.1107/S160053681202418X

**Published:** 2012-05-31

**Authors:** Shahedeh Tayamon, Thahira Begum S. A. Ravoof, Mohamed Ibrahim Mohamed Tahir, Karen A. Crouse, Edward R. T. Tiekink

**Affiliations:** a Department of Chemistry, Universiti Putra Malaysia, 43400 Serdang, Malaysia; b Department of Chemistry, University of Malaya, 50603 Kuala Lumpur, Malaysia

## Abstract

In the title disulfide, C_16_H_18_S_2_, the mol­ecule is twisted about the central S—S bond [the C—S—S—C torsion angle = 93.24 (7)°] and the dihedral angle between the benzene rings is 72.84 (7)°, indicating an almost orthogonal relationship; the methyl groups are orientated to the same side of the mol­ecule. The crystal packing features C—H⋯.π inter­actions which consolidate a three-dimensional architecture.

## Related literature

For background to the coordination chemistry of dithio­carbazate derivatives, see: Crouse *et al.* (2004 ▶); Ravoof *et al.* (2010 ▶). For the synthesis and methodology, see: Tarafder *et al.* (2000 ▶). For the structure of bis­(benz­yl)disulfide, see: van Dijk & Visser (1971 ▶).
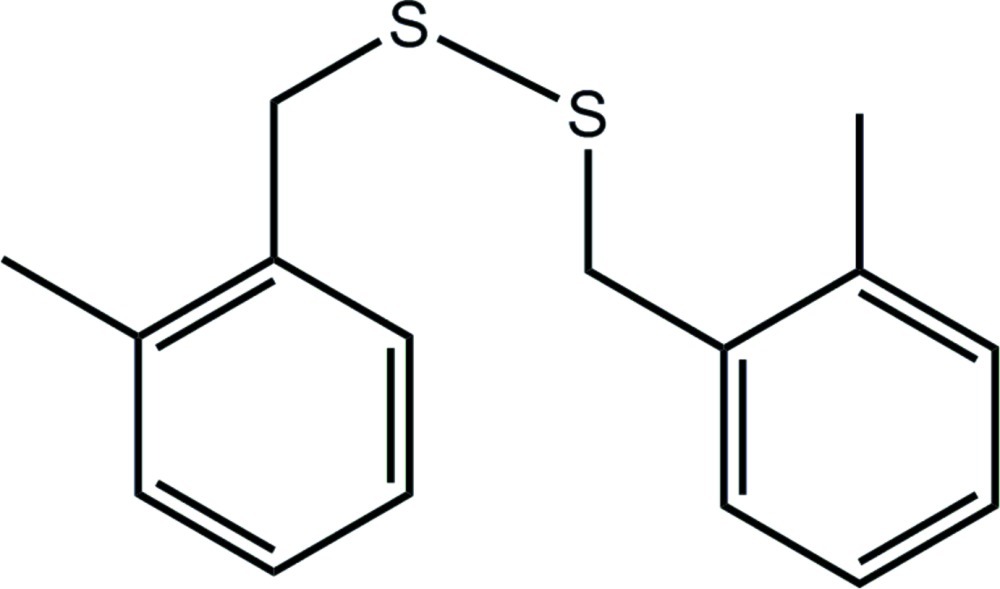


## Experimental

### 

#### Crystal data

C_16_H_18_S_2_
*M* *_r_* = 274.42
Monoclinic, 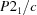

*a* = 10.3640 (4) Å
*b* = 7.6408 (3) Å
*c* = 18.1106 (7) Å
β = 91.099 (3)°
*V* = 1433.90 (10) Å^3^
*Z* = 4
Cu *K*α radiation
μ = 3.18 mm^−1^
*T* = 100 K
0.56 × 0.38 × 0.21 mm

#### Data collection

Oxford Diffraction Xcalibur Eos Gemini diffractometer
Absorption correction: multi-scan (*CrysAlis PRO*; Agilent, 2011 ▶) *T*_min_ = 0.299, *T*_max_ = 0.513
9993 measured reflections
2773 independent reflections
2672 reflections with *I* > 2σ(*I*)
*R*_int_ = 0.023

#### Refinement

*R*[*F* ^2^ > 2σ(*F* ^2^)] = 0.035
*wR*(*F* ^2^) = 0.096
*S* = 1.06
2773 reflections
165 parameters
H-atom parameters constrained
Δρ_max_ = 0.41 e Å^−3^
Δρ_min_ = −0.22 e Å^−3^

### 

Data collection: *CrysAlis PRO* (Agilent, 2011 ▶); cell refinement: *CrysAlis PRO*; data reduction: *CrysAlis PRO*; program(s) used to solve structure: *SHELXS97* (Sheldrick, 2008 ▶); program(s) used to refine structure: *SHELXL97* (Sheldrick, 2008 ▶); molecular graphics: *ORTEP-3* (Farrugia, 1997 ▶) and *DIAMOND* (Brandenburg, 2006 ▶); software used to prepare material for publication: *publCIF* (Westrip, 2010 ▶).

## Supplementary Material

Crystal structure: contains datablock(s) global, I. DOI: 10.1107/S160053681202418X/hb6818sup1.cif

Structure factors: contains datablock(s) I. DOI: 10.1107/S160053681202418X/hb6818Isup2.hkl

Supplementary material file. DOI: 10.1107/S160053681202418X/hb6818Isup3.cml

Additional supplementary materials:  crystallographic information; 3D view; checkCIF report

## Figures and Tables

**Table 1:** Hydrogen-bond geometry (Å, °) *Cg*1 and *Cg*2 are the centroids of the C2–C7 and C10–C15 rings, respectively.

*D*—H⋯*A*	*D*—H	H⋯*A*	*D*⋯*A*	*D*—H⋯*A*
C1—H1*B*⋯*Cg*1^i^	0.99	2.91	3.4605 (16)	116
C16—H16*B*⋯*Cg*2^ii^	0.98	2.85	3.7392 (18)	151
C16—H16*C*⋯*Cg*1^iii^	0.98	2.97	3.7764 (18)	140

Symmetry codes: (i) 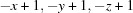; (ii) 
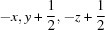
; (iii) 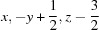.

**Fig. 1.**
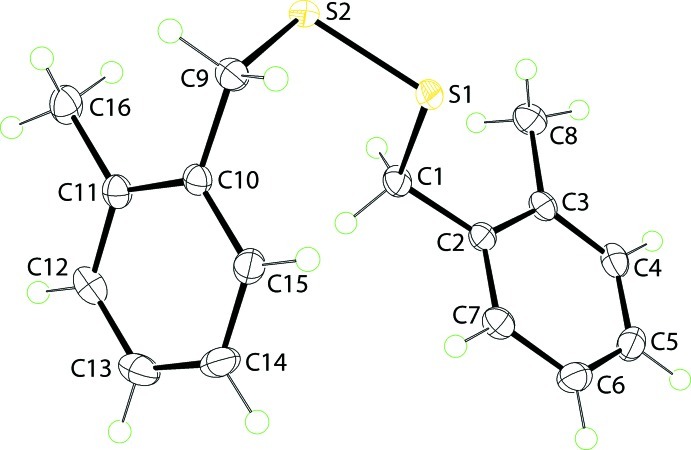
The molecular structure of (I) showing the atom-labelling scheme and displacement ellipsoids at the 50% probability level.

**Fig. 2.**
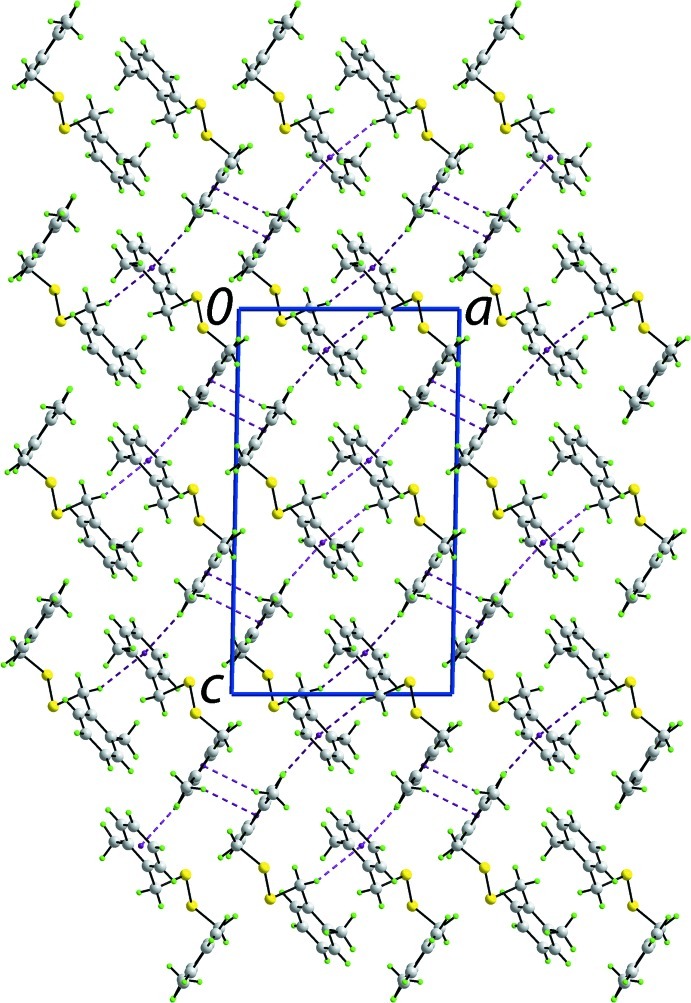
A view in projection down the b axis of the unit-cell contents for (I). The C—H···π interactions are shown as purple dashed lines.

## supplementary crystallographic information

### Comment

Our interest in investigating the coordination properties of ligands containing the H—N—C═S moiety and our desire to expand the study of this class of biologically important compounds has lead us to synthesize series of related ligands (Tarafder *et al.*, 2000; Crouse *et al.*, 2004; Ravoof *et al.*, 2010). The title compound, [(2-methyl)benzyl]disulfide, (I), was obtained during the attempt to prepare the phenyl hydrazine analogue of *S*-benzyldithiocarbazate.

In (I), Fig. 1, the molecule is twisted about the central S1—S2 bond as seen in the value of the C1—S1—S2—C9 torsion angle of 93.24 (7) Å. The dihedral between the benzene rings is 72.84 (7)°, indicating an almost orthogonal relationship, and the methyl groups are orientated to the same side of the molecule. The overall conformation in (I) contrasts that found in the parent compound, bis(benzyl)disulfide (van Dijk & Visser, 1971), which adopts an open conformation with both phenyl rings directed away from the sulfur atoms. In (I), the S1-bound benzyl is directed away having an *anti* disposition [the S2—S1—C1—C2 torsion angle is 176.23 (9)°] whereas the S2-bound benzyl residue in bis(benzyl)disulfide (van Dijk & Visser, 1971) has a *syn* conformation [S1—C1—C2—C7 = 91.90 (14)°].

The crystal packing is dominated by C—H···.π interactions, Table 1, which consolidates a three-dimensional architecture, Fig. 2.

### Experimental

[(2-Methyl)benzyl]disulfide was isolated as a by-product from the synthesis of the phenylhydrazine analog of *S*-benzyldithiocarbazate (Tarafder *et al.*, 2000). Potassium hydroxide (0.2 mol, 11.2 g) was completely dissolved in absolute ethanol (70 ml) and phenylhydrazine (0.2 mol, 21.6 g) was added to the solution cooled in an ice-salt bath producing a dark-yellow solution. Carbon disulfide (0.2 mol, 15.2 g) was added drop-wise with constant stirring over one hour. 2-Methylbenzyl chloride (0.1 mol, 13.2 ml) was then added drop-wise with vigorous stirring. The temperature of reaction was maintained below 278 K. The high yield yellow-white product was filtered and dried in a dessicator over anhydrous silica gel, dissolved in absolute ethanol and kept in a freezer. A few colourless blocks were harvested on the third day and washed with cold n-hexane.

### Refinement

Carbon-bound H-atoms were placed in calculated positions (C—H = 0.95 to 0.99 Å) and were included in the refinement in the riding model approximation with *U*_iso_(H) set to 1.2 to 1.5*U*_equiv_(C).

### Crystal data

**Table:**

C_16_H_18_S_2_	*F*(000) = 584
*M**_r_* = 274.42	*D*_x_ = 1.271 Mg m^−^^3^
Monoclinic, *P*2_1_/*c*	Cu *K*α radiation, λ = 1.54180 Å
Hall symbol: -P 2ybc	Cell parameters from 5536 reflections
*a* = 10.3640 (4) Å	θ = 4–71°
*b* = 7.6408 (3) Å	µ = 3.18 mm^−^^1^
*c* = 18.1106 (7) Å	*T* = 100 K
β = 91.099 (3)°	Block, colourless
*V* = 1433.90 (10) Å^3^	0.56 × 0.38 × 0.21 mm
*Z* = 4	

### Data collection

**Table:**

Oxford Diffraction Xcalibur Eos Gemini diffractometer	2773 independent reflections
Radiation source: fine-focus sealed tube	2672 reflections with *I* > 2σ(*I*)
Graphite monochromator	*R*_int_ = 0.023
Detector resolution: 16.1952 pixels mm^-1^	θ_max_ = 71.6°, θ_min_ = 4.3°
ω scans	*h* = −12→12
Absorption correction: multi-scan (*CrysAlis PRO*; Agilent, 2011)	*k* = −8→9
*T*_min_ = 0.299, *T*_max_ = 0.513	*l* = −21→22
9993 measured reflections	

### Refinement

**Table:**

Refinement on *F*^2^	Primary atom site location: structure-invariant direct methods
Least-squares matrix: full	Secondary atom site location: difference Fourier map
*R*[*F*^2^ > 2σ(*F*^2^)] = 0.035	Hydrogen site location: inferred from neighbouring sites
*wR*(*F*^2^) = 0.096	H-atom parameters constrained
*S* = 1.06	*w* = 1/[σ^2^(*F*_o_^2^) + (0.0629*P*)^2^ + 0.5578*P*] where *P* = (*F*_o_^2^ + 2*F*_c_^2^)/3
2773 reflections	(Δ/σ)_max_ = 0.001
165 parameters	Δρ_max_ = 0.41 e Å^−^^3^
0 restraints	Δρ_min_ = −0.22 e Å^−^^3^

### Special details

**Table:**

Geometry. All s.u.'s (except the s.u. in the dihedral angle between two l.s. planes) are estimated using the full covariance matrix. The cell s.u.'s are taken into account individually in the estimation of s.u.'s in distances, angles and torsion angles; correlations between s.u.'s in cell parameters are only used when they are defined by crystal symmetry. An approximate (isotropic) treatment of cell s.u.'s is used for estimating s.u.'s involving l.s. planes.
Refinement. Refinement of *F*^2^ against ALL reflections. The weighted *R*-factor *wR* and goodness of fit *S* are based on *F*^2^, conventional *R*-factors *R* are based on *F*, with *F* set to zero for negative *F*^2^. The threshold expression of *F*^2^ > 2σ(*F*^2^) is used only for calculating *R*-factors(gt) *etc*. and is not relevant to the choice of reflections for refinement. *R*-factors based on *F*^2^ are statistically about twice as large as those based on *F*, and *R*- factors based on ALL data will be even larger.

### Fractional atomic coordinates and isotropic or equivalent isotropic displacement parameters (Å^2^)

**Table:**

	*x*	*y*	*z*	*U*_iso_*/*U*_eq_	
S1	0.19303 (3)	0.79536 (5)	0.531391 (19)	0.01839 (13)	
S2	0.16204 (3)	0.97650 (5)	0.450468 (19)	0.01857 (13)	
C1	0.32793 (14)	0.6661 (2)	0.49591 (8)	0.0197 (3)	
H1A	0.3014	0.6058	0.4497	0.024*	
H1B	0.4019	0.7435	0.4853	0.024*	
C2	0.36554 (14)	0.5340 (2)	0.55430 (8)	0.0175 (3)	
C3	0.45586 (14)	0.5739 (2)	0.61072 (8)	0.0190 (3)	
C4	0.48816 (15)	0.4427 (2)	0.66150 (8)	0.0231 (3)	
H4	0.5498	0.4670	0.6996	0.028*	
C5	0.43266 (16)	0.2782 (2)	0.65765 (9)	0.0252 (4)	
H5	0.4571	0.1908	0.6925	0.030*	
C6	0.34159 (17)	0.2409 (2)	0.60306 (10)	0.0254 (3)	
H6	0.3022	0.1287	0.6007	0.030*	
C7	0.30838 (15)	0.3688 (2)	0.55173 (8)	0.0216 (3)	
H7	0.2457	0.3434	0.5143	0.026*	
C8	0.51809 (16)	0.7523 (2)	0.61716 (10)	0.0258 (4)	
H8A	0.5715	0.7574	0.6623	0.039*	
H8B	0.4509	0.8424	0.6191	0.039*	
H8C	0.5722	0.7729	0.5742	0.039*	
C9	0.03670 (14)	0.8731 (2)	0.39237 (8)	0.0197 (3)	
H9A	−0.0361	0.8389	0.4239	0.024*	
H9B	0.0039	0.9600	0.3561	0.024*	
C10	0.08223 (14)	0.71442 (19)	0.35152 (8)	0.0168 (3)	
C11	0.15632 (14)	0.7299 (2)	0.28736 (8)	0.0187 (3)	
C12	0.19310 (14)	0.5778 (2)	0.25128 (9)	0.0232 (3)	
H12	0.2407	0.5869	0.2071	0.028*	
C13	0.16219 (16)	0.4131 (2)	0.27805 (9)	0.0265 (4)	
H13	0.1895	0.3111	0.2528	0.032*	
C14	0.09121 (16)	0.3982 (2)	0.34191 (10)	0.0262 (4)	
H14	0.0706	0.2861	0.3610	0.031*	
C15	0.05054 (15)	0.5485 (2)	0.37768 (9)	0.0209 (3)	
H15	0.0002	0.5382	0.4208	0.025*	
C16	0.19659 (16)	0.9069 (2)	0.25888 (9)	0.0263 (4)	
H16A	0.2582	0.9603	0.2939	0.039*	
H16B	0.1205	0.9822	0.2534	0.039*	
H16C	0.2373	0.8930	0.2108	0.039*	

### Atomic displacement parameters (Å^2^)

**Table:**

	*U*^11^	*U*^22^	*U*^33^	*U*^12^	*U*^13^	*U*^23^
S1	0.0218 (2)	0.0188 (2)	0.0146 (2)	0.00273 (13)	0.00327 (14)	−0.00021 (12)
S2	0.0244 (2)	0.0138 (2)	0.0175 (2)	−0.00018 (13)	−0.00079 (14)	−0.00113 (12)
C1	0.0196 (7)	0.0225 (8)	0.0173 (7)	0.0042 (6)	0.0039 (5)	−0.0003 (6)
C2	0.0176 (7)	0.0187 (8)	0.0164 (7)	0.0027 (6)	0.0046 (5)	−0.0009 (5)
C3	0.0176 (7)	0.0222 (8)	0.0175 (7)	0.0020 (6)	0.0036 (5)	−0.0031 (6)
C4	0.0223 (7)	0.0301 (9)	0.0169 (7)	0.0060 (6)	0.0009 (6)	−0.0017 (6)
C5	0.0297 (8)	0.0245 (9)	0.0217 (8)	0.0077 (6)	0.0065 (6)	0.0058 (6)
C6	0.0290 (8)	0.0188 (8)	0.0285 (8)	−0.0014 (6)	0.0074 (7)	0.0007 (6)
C7	0.0206 (7)	0.0231 (8)	0.0211 (7)	0.0001 (6)	0.0019 (6)	−0.0029 (6)
C8	0.0257 (8)	0.0254 (9)	0.0263 (8)	−0.0030 (7)	−0.0003 (6)	−0.0047 (7)
C9	0.0194 (7)	0.0202 (8)	0.0194 (7)	0.0015 (6)	−0.0010 (5)	−0.0025 (6)
C10	0.0167 (7)	0.0182 (8)	0.0155 (7)	0.0006 (5)	−0.0024 (5)	−0.0006 (5)
C11	0.0167 (7)	0.0221 (8)	0.0171 (7)	−0.0019 (6)	−0.0023 (5)	−0.0001 (6)
C12	0.0186 (7)	0.0313 (9)	0.0196 (7)	0.0003 (6)	0.0000 (6)	−0.0070 (6)
C13	0.0249 (8)	0.0229 (9)	0.0316 (9)	0.0054 (6)	−0.0061 (6)	−0.0096 (7)
C14	0.0306 (8)	0.0151 (8)	0.0325 (9)	−0.0022 (6)	−0.0087 (7)	0.0021 (6)
C15	0.0228 (7)	0.0216 (8)	0.0184 (7)	−0.0031 (6)	−0.0017 (6)	0.0023 (6)
C16	0.0305 (8)	0.0281 (9)	0.0203 (8)	−0.0084 (7)	0.0024 (6)	0.0026 (6)

### Geometric parameters (Å, º)

**Table:**

S1—C1	1.8377 (15)	C8—H8C	0.9800
S1—S2	2.0368 (5)	C9—C10	1.501 (2)
S2—C9	1.8349 (15)	C9—H9A	0.9900
C1—C2	1.508 (2)	C9—H9B	0.9900
C1—H1A	0.9900	C10—C15	1.395 (2)
C1—H1B	0.9900	C10—C11	1.410 (2)
C2—C7	1.395 (2)	C11—C12	1.390 (2)
C2—C3	1.406 (2)	C11—C16	1.509 (2)
C3—C4	1.397 (2)	C12—C13	1.389 (3)
C3—C8	1.511 (2)	C12—H12	0.9500
C4—C5	1.384 (2)	C13—C14	1.387 (3)
C4—H4	0.9500	C13—H13	0.9500
C5—C6	1.383 (3)	C14—C15	1.388 (2)
C5—H5	0.9500	C14—H14	0.9500
C6—C7	1.387 (2)	C15—H15	0.9500
C6—H6	0.9500	C16—H16A	0.9800
C7—H7	0.9500	C16—H16B	0.9800
C8—H8A	0.9800	C16—H16C	0.9800
C8—H8B	0.9800		
			
C1—S1—S2	102.94 (5)	H8B—C8—H8C	109.5
C9—S2—S1	102.71 (5)	C10—C9—S2	113.87 (10)
C2—C1—S1	107.61 (10)	C10—C9—H9A	108.8
C2—C1—H1A	110.2	S2—C9—H9A	108.8
S1—C1—H1A	110.2	C10—C9—H9B	108.8
C2—C1—H1B	110.2	S2—C9—H9B	108.8
S1—C1—H1B	110.2	H9A—C9—H9B	107.7
H1A—C1—H1B	108.5	C15—C10—C11	119.47 (14)
C7—C2—C3	119.79 (14)	C15—C10—C9	119.22 (14)
C7—C2—C1	118.60 (14)	C11—C10—C9	121.31 (13)
C3—C2—C1	121.61 (14)	C12—C11—C10	118.41 (14)
C4—C3—C2	118.07 (15)	C12—C11—C16	120.55 (14)
C4—C3—C8	120.01 (14)	C10—C11—C16	121.04 (14)
C2—C3—C8	121.92 (14)	C13—C12—C11	121.77 (15)
C5—C4—C3	121.66 (15)	C13—C12—H12	119.1
C5—C4—H4	119.2	C11—C12—H12	119.1
C3—C4—H4	119.2	C14—C13—C12	119.66 (15)
C4—C5—C6	120.03 (15)	C14—C13—H13	120.2
C4—C5—H5	120.0	C12—C13—H13	120.2
C6—C5—H5	120.0	C13—C14—C15	119.47 (15)
C5—C6—C7	119.40 (16)	C13—C14—H14	120.3
C5—C6—H6	120.3	C15—C14—H14	120.3
C7—C6—H6	120.3	C14—C15—C10	121.18 (15)
C6—C7—C2	121.02 (15)	C14—C15—H15	119.4
C6—C7—H7	119.5	C10—C15—H15	119.4
C2—C7—H7	119.5	C11—C16—H16A	109.5
C3—C8—H8A	109.5	C11—C16—H16B	109.5
C3—C8—H8B	109.5	H16A—C16—H16B	109.5
H8A—C8—H8B	109.5	C11—C16—H16C	109.5
C3—C8—H8C	109.5	H16A—C16—H16C	109.5
H8A—C8—H8C	109.5	H16B—C16—H16C	109.5
			
C1—S1—S2—C9	93.24 (7)	S1—S2—C9—C10	−68.30 (11)
S2—S1—C1—C2	176.23 (9)	S2—C9—C10—C15	102.14 (14)
S1—C1—C2—C7	91.90 (14)	S2—C9—C10—C11	−77.83 (16)
S1—C1—C2—C3	−87.97 (15)	C15—C10—C11—C12	1.4 (2)
C7—C2—C3—C4	1.9 (2)	C9—C10—C11—C12	−178.67 (13)
C1—C2—C3—C4	−178.24 (13)	C15—C10—C11—C16	−177.79 (14)
C7—C2—C3—C8	−178.44 (14)	C9—C10—C11—C16	2.2 (2)
C1—C2—C3—C8	1.4 (2)	C10—C11—C12—C13	−2.0 (2)
C2—C3—C4—C5	−0.7 (2)	C16—C11—C12—C13	177.16 (14)
C8—C3—C4—C5	179.60 (15)	C11—C12—C13—C14	0.9 (2)
C3—C4—C5—C6	−0.8 (2)	C12—C13—C14—C15	0.9 (2)
C4—C5—C6—C7	1.1 (2)	C13—C14—C15—C10	−1.5 (2)
C5—C6—C7—C2	0.1 (2)	C11—C10—C15—C14	0.4 (2)
C3—C2—C7—C6	−1.6 (2)	C9—C10—C15—C14	−179.62 (14)
C1—C2—C7—C6	178.51 (14)		

### Hydrogen-bond geometry (Å, º)

Cg1 and Cg2 are the centroids of the C2–C7 and C10–C15 rings, respectively.

**Table:**

*D*—H···*A*	*D*—H	H···*A*	*D*···*A*	*D*—H···*A*
C1—H1*B*···*Cg*1^i^	0.99	2.91	3.4605 (16)	116
C16—H16*B*···*Cg*2^ii^	0.98	2.85	3.7392 (18)	151
C16—H16*C*···*Cg*1^iii^	0.98	2.97	3.7764 (18)	140

Symmetry codes: (i) −*x*+1, −*y*+1, −*z*+1; (ii) −*x*, *y*+1/2, −*z*+1/2; (iii) *x*, −*y*+1/2, *z*−3/2.
